# The factors associated with the burnout syndrome and fatigue in Cypriot nurses: a census report

**DOI:** 10.1186/1471-2458-12-457

**Published:** 2012-06-20

**Authors:** Vasilios Raftopoulos, Andreas Charalambous, Michael Talias

**Affiliations:** 1 Mediterranean Research Centre for Public Health and Quality of Care, Cyprus University of Technology, 215 Paleos dromos lefkosias lemesou, Nicosia, Cyprus; 2 Research Center for Oncology and Palliative Care, Cyprus University of Technology, 215 Paleos dromos lefkosias lemesou, Nicosia, Cyprus; 3 Open University of Cyprus, Nicosia, Cyprus

**Keywords:** Burnout syndrome, Nurses, Fatigue, Maslach’s burnout scale

## Abstract

**Background:**

Fatigue and burnout are two concepts often linked in the literature. However, regardless of their commonalities they should be approached as distinct concepts. The current and ever-growing reforms regarding the delivery of nursing care in Cyprus, stress for the development of ways to prevent burnout and effectively manage fatigue that can result from working in stressful clinical environments.

**Methods:**

To explore the factors associated with the burnout syndrome in Cypriot nurses working in various clinical departments. A random sampling method taking into account geographical location, specialty and type of employment has been used.

**Results:**

A total of 1,482 nurses (80.4% were females) working both in the private and public sectors completed and returned an anonymous questionnaire that included several aspects related to burnout; the MBI scale, questions related to occupational stress, and questions pertaining to self reported fatigue. Two-thirds (65.1%) of the nurses believed that their job is stressful with the majority reporting their job as stressful being female nurses (67.7%). Twelve point eight percent of the nurses met Maslach’s criteria for burnout. The prevalence of fatigue in nurses was found 91.9%. The prevalence of fatigue was higher in females (93%) than in males (87.5%) (p = 0.003). As opposed to the burnout prevalence, fatigue prevalence did not differ among the nursing departments (p = 0.166) and among nurses with a different marital status (p = 0.553). Burnout can be associated adequately knowing if nurses find their job stressful, their age, the level of emotional exhaustion and depersonalization. It has been shown that the fatigue may be thought of as a predictor of burnout, but its influence is already accounted by emotional exhaustion and depersonalization.

**Conclusion:**

The clinical settings in Cyprus appear as stress generating environment for nurses. Nurses working both in the private and public sector appear to experience low to severe burnout. Self-reported fatigue interferes to the onset of emotional exhaustion and depersonalization.

## Background

The impact of stress and its consequences has been at the center of many healthcare studies in the past [1]. The constant interaction between professional standards, personal ego integrity and patient needs within the therapeutic relationship often leave the nurse vulnerable to stress, fatigue, and burnout.

For nursing, the topic of stress has received its’ greater attention in the form of exploring the effects of the “burnout syndrome” (BOS). Nurses are more susceptible to experiencing burnout than some of the other healthcare professions because of the implicit relationship of job stress to burnout. There have been many studies trying to verify the relationship between stress and burnout in various clinical settings [2-4] however; little light has been shed on specific associations and inter-relationships between the two concepts.

The burnout syndrome refers to a situation in which workers appear disconnected from their job and everything seems to be senseless and any effort or activity, meaningless. In 1974, Freudenberger coined the term “burnout” to describe workers’ reactions to the chronic stress commonly found in occupations involving numerous interpersonal interactions [5]. Burnout is typically conceptualized as a syndrome characterized by emotional exhaustion, depersonalization, and reduced personal accomplishment [5]. The term “burnout syndrome”, mainly applied to the caring professions, defines the breakdown of energy resources and adaptability as a reaction to chronic stress [6-8].

There has been much research on burnout in nurses, presumably because of the intense nature of their contact with patients/clients [9]. However, studies undertaken in different groups of nurses show variations in the expressed levels of burnout. Variation exists also in terms of the consequences of burnout [10]. Burnout can be manifested as psychological distress, somatic complaints, alcohol and drug abuse for healthcare workers [11,12]. In this light Melchior et al. [13] assert that burnout has been related virtually to every symptom due to the ambiguity surrounding this concept. This can be attributed to the varying responses of people towards burnout.

In relation to the contributing factors to nurses’ burnout Schaufeli [14] and Duquette et al. [15] assert that based on the available evidence there are various levels of correlations. However, both researchers assert that there is sufficient evidence to show that age, work pressure, role confusion, less hardiness, passive coping style and limited social support can negatively influence burnout in nurses. Several studies have indicated that the presence of social resources can contribute to low levels of burnout [16,17]. These contributing factors have also been identified earlier by Maslach et al. [18].

Burnout and fatigue are related but conceptually are different constructs. Therefore burnout is conceptualized as a work related condition and fatigue as a more general condition. An interesting theorization of fatigue comes from Valent [19] who asserts that fatigue occurs when one cannot rescue or save the individual from harm and results in guilt and distress. On the other hand, fatigue according to Shen et al. [20] refers to an overwhelming sense of tiredness, lack of energy, and a feeling of exhaustion associated with impaired physical and/or cognitive functioning.

In 1992 a related term to fatigue was introduced in the literature that of “compassion fatigue” [21]. Yoder [22] asserts that the term referred to situations in which nurses had either turned off their own feelings or experienced helplessness and anger in response to the stress they feel watching patients go through devastating illnesses or trauma.

Studies in the wider working population have shown that fatigue can be associated with sickness absence and work disability [21,23]. For the nursing population the studies have demonstrated that long working hours, rotating shifts and night shifts can lead nurses to fatigue. The effects of fatigue include but are not limited to poor performance, errors in clinical practice, and prolonged fatigue may lead to burnout. Existing evidence support that the healthcare workers’ performance on tasks requiring vigilance, attention to detail, or which are long in duration may be particularly susceptible to fatigue-related consequences [24,25].

On a financial basis, for hospitals, burnout, stress and fatigue can be costly leading to increased employee tardiness, absenteeism, turnover, decreased performance, and difficulty in recruiting and retaining staff [26,27]. Based on the preceding studies, it seems unlikely that healthcare organizations with high levels of burnout among health professionals could achieve the performance characteristics such as patient-centeredness as a strategy to improve quality of care [28,29].

### Healthcare context in Cyprus

Cyprus has a mixed health care system, which is in transition to a National Health Care System. Being a mixed system, it means that the public has the option to receive care either by a public or a private provider. However, there appear to be major discrepancies between public and private hospitals. Public hospitals are responsible for providing primary (primary care is the first point of contact a person has with the health system), secondary (provision of acute and specialist services, treating conditions which normally cannot be dealt with by primary care specialists or which are brought in as an emergency) and tertiary care (provision of specialized consultative care, usually on referral from primary or secondary medical care personnel), where as the private hospitals are confined to provide secondary care, and limited preventive services. The public hospitals are faced up with a challenge; how to meet the increasing demand for health care without an adequate increase of resources [30-32].

The private hospitals’ operation is contingent on market incentives. Because private hospitals are not subsidised and depend on income from clients/users, it can be argued that they are more inclined than public hospitals to provide quality services and to be concerned about client/user satisfaction [33]. The majority of the population (95%) is entitled to either free medical care, or to publicly provided healthcare at reduced cost coverage. The remaining percentage of the population seeks health care services from the private establishments (i.e. private hospitals, clinics).

Nursing personnel comprise the largest group of healthcare workers employed both by public and private hospitals. The nursing education is Cyprus is provided on a bachelor’s level, requiring 4 years of education and training. Nursing education is nowadays provided solely by public and private universities. Previously, nurses were educated on a diploma basis requiring only 3 years of education. Registered nurses under the new act (released on 2012) are required to renew their practice license every four years based on specific criteria in relation to lifelong learning.

However, as a result of ongoing change, due to the introduction of a National Healthcare System, nurses face challenges requiring them to balance high-quality care with lower costs [33]. The consequence of this on nurses has been considerable and far-reaching [16]. With less nurses to care for patients, their workload significantly increased. Overall, stress levels also increased when more patients had to be processed in the same number of hours and patient turnover is faster than in the past [34,35].

Taking the above into consideration and keeping in mind that the phenomenon of burnout has not been examined within the population of nurses in the Cypriot Healthcare context, a research study was undertaken to investigate the burnout syndrome within this population. Furthermore, there is a gap in the international literature in relation to the study of the prevalence of burnout and fatigue among nurses, demonstrating the need for further research in this field.

This study was designed to explore the factors associated with the burnout syndrome in Cypriot nurses who work in the private and public healthcare sectors.

The research questions posed by this study were the following:

1. What is the point prevalence of burnout syndrome in Cypriot nurses?

2. Which factors are associated with burnout syndrome in Cypriot nurses?

3. What is the difference in burnout syndrome between Cypriot nurses working in the private and the public sector?

4. Is there an association between fatigue and burnout?

## Methods

Subjects were selected based on their willingness to participate and on the following pre-determined inclusion criterion: being a registered nurse and working in the public or private sectors. Potential participants were recruited on the basis of their availability. The researchers had personal meetings with the potential participants during which they provided detailed explanations on the purpose and aim of the study as well as what was actually expected of them. An informed consent was obtained from those who agreed to participate in the study. The participants were then asked to complete the study’s questionnaire. In Cyprus there are approximately 2,800 nurses working in the public and private sector. The questionnaire was administered to a convenience sample of nurses during the provision of a training program for upgrading from diploma to bachelor level in nursing, provided by the Nursing Department of the Cyprus University of Technology. The vast majority of the nurses in Cyprus participated in this 2-year program. The questionnaires were distributed in the classes during breaks and have been collected during the same period in order to enhance response rate. Thus taking into account geographical location, specialty and type of employment, 1,500 nurses working both in the private and public sectors were approached. In total, one thousand four hundred and eighty questionnaires were completed and returned (response rate 98.6%). Incomplete questionnaires were excluded from the analysis. Data collection was conducted in the period between September 2010 and May 2011.

### Instrument

This survey was based on an anonymous self-administered questionnaire, which was distributed to the nurses and was returned anonymously in the provided boxes allocated at the nurses’ working places. This was considered necessary in order to assure confidentiality. The face validity of the questionnaire was explicitly assessed through feedback from a panel of experts (researchers, health-care professionals, and academics) who reviewed the questionnaire and confirmed its final version with minor wording changes. According to Lynn [36], the minimum number of experts required is five. The expert review and the panel discussion took place at the Cyprus University of Technology. Initially, the experts were asked to respond independently to a questionnaire that was developed for the assessment of the questionnaire. They were asked to rate the clarity, the concreteness, the centrality, and the importance of each item using a three-point rating scale (1 = “not clear”, 2 = “clear”, and 3 = “very clear”). The items were considered adequate if there was > 90% agreement, based on the use of kappa statistics [37]. The feedback resulted in suggestions that contributed to the improvement of the questionnaire.

The first part of the questionnaire included sociodemographic information such as age, gender, education, marital status, years of work experience as a registered nurse and the second part included the MBI scale and general questions on nurses’ perceived quality of care provided. Several instruments have been developed in the course of time to assess the burnout in the healthcare professionals such as the instruments developed by Pines and colleagues [38], Jones [39] and Matthiesen [40] however the most prominent is the Maslach’s Burnout Inventory (MBI) developed by Maslach and Jackson [9].

The MBI scale is a tool for detecting and measuring the severity of the burnout syndrome. It is a 22-item instrument that assesses the degree of burnout in terms of three subscales: emotional exhaustion (EE), feelings of being emotionally exhausted and lack of energy, depersonalization (DP), and feelings of impersonal response towards recipients of the service and lack of personal accomplishment (PA), feeling of incompetence. This instrument has already been validated in the Greek language [41] and has been recently tested on a sample of oncology nurses and physicians in Greece [32] and in a sample of physiotherapists in Cyprus [42]. High scores in the EE or DP scales or low scores in the personal accomplishment scale indicate high levels of burnout. For the purpose of the current study, scores within individual burnout domains were used both as continuous variables and categorized into low, intermediate and high scores using the cutoffs suggested by Maslach et al. [43].

In the current research the reliability analysis revealed that Cronbach’s alpha were 0.83, 0.76 and 0.70 for the EE PA DP subscales respectively, values that are comparable to those found in the validation study [32]. The EE subscale contains 9 items, PA 8 items and DP 5 items. It is of note that a high coefficient alpha does not always mean a high degree of internal consistency when the scale has many items, e.g. more than about 15. This is because alpha is highly sensitive to the number of items in the scale. We acknowledge that the marginal Cronbach’s alphas in the subscales PA and DP have been also documented in other studies. The MBI is a well established scale that has been validated in Greek-Cypriot sample. The validation study of the Greek version of the MBI revealed the following: EE alpha = 0.84, PA alpha = 0.71 and DP alpha = 0.55. In a similar research with oncology nurses in Greece alpha scores were as follows: EE alpha = 0.85, PA alpha = 0.78 and DP alpha = 0.70 and in a research with Cypriots nurses EE alpha = 0.84, PA alpha = 0.62 and DP alpha = 0.58 [42]. In the Soler et al. study [44] Cronbach’s alpha for depersonalization ranged across the participant countries from 0.46 to 0.91 and for personal accomplishment from 0.67 to 0.85 [44-47]. Compared to the preceding validation studies the instrument has marginally acceptable reliability scores. As a result the instrument appears reliable for the Greek-Cypriot nursing population.

The Nursing Department’s Ethics Committee of the Cyprus University of Technology granted ethical approval for this study.

## Statistical analysis

All of the items were coded and scored, and the completed questionnaires were included in the data analysis set. IBM SPSS statistics 19 was used to analyze the data. The chi-square test was used to explore the existence of a statistically significant relationship between the categorical variables. The *t*-test was used to assess whether the means of two groups were statistically different from each other, while for the comparison of the aforementioned scores between three or more groups analysis of variance (ANOVA) was used (the dependent variable is assumed to be normally distributed in the populations). P values < 0.05 were considered to be statistically significant, unless otherwise stated. Internal consistency of the MBI scale was assessed by calculating Cronbach’s alpha [48].

We estimated various multilevel logistic regression models with cross effects to investigate the connection between having burnout and the factors associated with the burnout syndrome such as feeling that their job is stressful, fatigue, age, gender, EE, DP, PA, geographical location and whether they work in the private or in the public sector. The multilevel model was undertaken using the statistical package lme4 of the R software, version 2.14.1 (http://cran.r-project.org). It was found that feeling that their job is stressful, age, EE, DP and the interaction of EE and DP were significant at the level of individuals and these were the fixed effects of the model. Taking into account the hierarchical structure of the data, we were allowed for random effects where we treated gender as a random effect due to unbalanced data (80.8% were females and 19.2% were males). We also assumed that the gender varied within the five geographical locations and that fatigue may have a random effect within gender.

## Results

One thousand four hundred and eighty two nurses participated in the study (80.4% were females). As seen in Table 1, more than two-thirds of the respondents were married. The majority of the respondents (96.6%) were employed in the public sector. There was a difference (p = 0.002) in the mean age between females (37.12 ± 10.32) and males (34.61 ± 10.50).

**Table 1:** Social and demographic characteristics of the sample

		**Total**		**Public sector**		**Private sector**
**Variable**	**N**	**%**	**N**	**%**	**N**	**%**
**Gender**						
Men	282	19.2	274	19.6	2	4.3
Women	1,189	80.8	1,123	80.4	45	95.7
**Marital status**						
Married	1,077	73.6	1,021	73.5	37	80.4
Single	257	17.5	248	17.8	3	6.5
Partner	50	3.4	49	3.5	1	2.1
Widower/widow	20	1.4	20	1.4	1	2.1
Divorced/Separated	60	4.1	53	3.8	4	8.9
**Working in**	1,455	98.5	1,408	96.8	47	3.2
Place						
Nicosia	783	52.8	719	51.1	41	87.2
Limassol	307	20.7	302	21.4	5	10.6
Pafos	127	8.6	127	9.0	-	-
Larnaca	161	10.9	156	11.1	1	2.2
Paralimni	104	7.0	104	7.4	-	-
**Department**						
Medical	109	7.6	106	7.6	-	-
Surgical	134	9.3	134	9.8	-	-
Emergency	142	9.9	142	10.3	-	-
ICU	121	8.4	121	8.8	-	-
Oncology	82	5.7	75	5.5	4	8.7
Operating theatre	88	6.1	87	6.3	-	-
Administration	38	2.6	34	2.5	1	2.2
Other	727	50.5	675	49.2	41	89.1
**Burned out**^**a**^	177	12.8	167	12.7	5	12.2
**Mean age ± sd (range)**	1,240	36.68 ± 10.39 (21–63)	1,173	36.38 ± 10.25 (21–60)	41	43.32 ± 11.40 (23–63)
**Mean working experience ± sd (range)**	1,444	14.53 ± 10.58 (1–41)	1,373	14.26 ± 10.45.(1–41)	45	20.87 ± 11.96 (1–41)
**Mean emotional exhaustion ± sd (range)**	1,331	22.15 ± 10.45 (0–54)	Table 3			
**Mean personal accomplishment ± sd (range)**	1,325	37.67 ± 6.58 (6–48)				
**Mean depersonalization ± sd (range)**	1,328	8.23 ± 6.02 (0–30)				

^a^Burnout as a dichotomous variable was defined as having a high DP score (>11) and high EE score (>31).

*sd*: standard deviation.

### Characteristics of burnout syndrome in Cypriot nurses

Almost two-thirds (65.1%) of the nurses believed that their job is stressful. Sixty seven point seven percent of female nurses as opposed to 55.5% of the male nurses (p < 0.001) reported that their job is stressful. Table 2 summarizes the mean scores computed for each of the three MBI subscales (EE, DP, PA) for the burned out and for the non burned out nurses. Twenty one point five percent of the participants were in the high EE range, 30.7% scored high in the PA section and 33% scored high in the DP section of the scale.

**Table 2:** Mean scores* in the MBI subscales

		**EE**			**DP**			**PA**	
	**Mean score**	**Mean age**	**Mean work experience**	**Mean score**	**Mean age**	**Mean work experience**	**Mean score**	**Mean age**	**Mean work experience**
**Non Burned out**									
**Low (95%CI)Correlations**	(N** = 688) 13.57(13.26–13.88)	(N = 590) 37.49(36.64–38.35)(r = −0.022)NS	(N = 677) 15.33(14.51–16.15)(r = −0.004)NS	(N = 546)2.49(2.36–2.62)	(N = 463) 38.47(37.52–39.42)(r = −0.09)p = 0.008	(N = 536) 16.42(15.52–17.32)(r = −0.06)p = 0.05	(N = 388) 44.35(44.15–44.55)	(N = 334) 40.24(39.09–41.39)(r = 0.10)p = 0.001	(N = 381) 18.20(17.10–19.29)(r = 0.11)P < 0.001
**Moderate (95%CI)Correlations**	(N = 409) 25.22(24.95–25.50)	(N = 340) 36.56(35.43–37.68)(r = −0.3)NS	(N = 401) 14.44(13.38–15.49)(r = −0.058)NS	(N = 390)7.76(7.62–7.90)	(N = 342) 36.39(35.30–37.49)(r = −0.05)NS	(N = 382) 13.59(12.55–14.64)(r = −0.06)NS	(N = 494) 38.79(38.64–38.95)	(N = 425) 36.84(35.83–37.85)(r = 0.02)NS	(N = 489) 14.49(13.54–15.45)(r = 0.05)NS
**High (95%CI)Correlations**	(N = 124) 36.82(35.73–37.91)	(N = 102) 34.51(33.36–35.66)(r = −0.065)NS	(N = 120) 14.17(12.29–16.05)(r = −0.045)NS	(N = 286) 14.31(13.95–14.67)	(N = 231) 35.55(34.17–36.93)(r = 0.02)NS	(N = 281) 12.63(11.68–13.58)(r = −0.01)NS	(N = 337) 30.03(29.47–30.59)	(N = 274) 34.00(32.91–35.08)(r = 0.06)NS	(N = 326) 11.89(10.83–12.94)(r = 0.07)NS
**Burned out**									
**Low (95%CI)Correlations**	-	-	-	-	-	-	(N = 20) 45.00 (42.24–47.76)	(N = 15) 37.67 (32.25–43.09)(r = 0.32)NS	(N = 19) 13.26 (8.16–18.36)(r = 0.02)NS
**Moderate (95%CI)Correlations**	-	-	-	-	-	-	(N = 65) 38.48 (38.13–38.83)	(N = 55) 34.76 (32.32–37.21)(r = 0.08)NS	(N = 64) 12.41 (10.08–14.73)(r = 0.08)NS
**High (95%CI)Correlations**	(N = 177) 38.13 (37.29–39.02)	(N = 154) 32.90 (31.55–34.26)(r = 0.215)p = 0.001	(N = 175) 10.53 (9.18–11.89)(r = 0.10)p = 0.05	(N = 177) 17,35 (16.66–18.04)	(N = 154) 32.90 (31.55–34.26)(r = 0.23)NS	(N = 175) 10.53 (9.18–11.89)(r = 0.28)NS	(N = 91) 29.29 (28.23–30.34)	N = 83 31.19 (21.52–32.87)(r = −0.01)NS	(N = 91) 9.13 (7.59–10.67)(r = −0.09)NS

The 95% CI have been calculated using the binomial Wald assumption (Brown et al. 2001).

*EE* (Emotional Exhaustion): high (≥31), moderate (21–30), low (≤20).

*PA* (Personal Accomplishment): high (≤35), moderate (41–36), low (≥42).

*DP* (Depersonalization): high (≥11), moderate (6–10), low (≤5).

Age range for the burned out: 21–60 and for the non burned out: 21–63.

Work experience for the burned out: 1–40 and for the non burned out: 1–41.

*Scores within individual burnout domains were used both as continuous variables and categorized into low, intermediate and high scores using the cutoffs suggested by Maslach et al.[8].

**The sample size does not total to 1,482.

*NS*: Non significant.

In total, 12.8% of the nurses met Maslach’s criteria for burnout [a high DP score (>11) and high EE score (>31)]. The point prevalence of burnout was:

(1) 12.7% for those who work in the public sector and 12.2% in the private sector (p = 0.580)

(2) 11.8% in females and 17.4% in males (p = 0.018)

(3) 12.5% in the partners, 14.8% in the divorced/separated, 11.5% in the married, 19.4% in the single (p = 0.007)

(4) 21.9% for those who work in the oncology departments, 17.5% in the operating theatre, 17.2% in the surgical, 15.9% in the emergency department, 14.5% in the ICU, 12.4% in the medical, 10.1% in the other departments and 2.9% in the Administration (p = 0.011)

(5) 16.8% for those who work in a department other than that of their choice (p < 0.001) compared to those who answered that it was their own choice (9.6%)

(6) 11.2% for those who stressed that the nursing job was their own choice, 17.3% for those who became nurses because of the circumstances and 12.2% for those who were empowered by significant others (p = 0.032).

Table 3 presents the MBI subscale scores by social and demographic characteristics. The mean DP score was significantly higher in men (p < 0.001). Gender, however, was not a significant variable for EE or the PA subscale. Conversely, females were more likely to suffer from burnout compared to males (OR = 1.582, 95%CI = 1.096–2.282). Marital status was not associated with MBI dimensions. The employment sector affected the level of PA (p < 0.001) with those who worked in the public sector having lower mean PA score. Furthermore, mean EE and DP scores were higher in those who replied that working in this department was not their own choice as opposed to the mean PA score that was higher in those who replied that working in this department was their own choice. The mean EE and DP scores indicate that burnout syndrome was significantly higher in the nurses who believed that their job was stressful. The nurses who claimed that their job was not their own choice but certain circumstances urged them to become nurses were found statistically significant more emotionally exhausted compared to those who answered that it was their own choice (p < 0.001) and to those who were encouraged from the significant others (p = 0.015). On the contrary PA mean score was higher to those who believed that it was their own choice as opposed to those who were encouraged from the significant others (p = 0.001) and was not their own choice but certain circumstances urged them to become nurses (p = 0.001). The results are similar for DP mean score as is was lower for those it was their own choice as opposed to those who were encouraged from the significant others (p < 0.001) and was not their own choice but certain circumstances urged them to become nurses (p = 0.009).

**Table 3:** **The associations between variables and the three burnout subscales (mean scores) explored using *t *-test and ANOVA tests as appropriate**

**Variable**	**EE mean (95% CI)**	**DP mean (95% CI)**	**PA mean (95% CI)**
**Gender**			
Men	21.52 (20.24–22.81)	10.15 (9.40–10.90)	37.14 (36.26–38.02)
Women	22.29 (21.68–22.89)	7.77 (7.43–8.11)	37.81 (37.44–38.19)
p value	NS	<0.001	NS
**Marital status**			
Cohabit	23.38 (20.49–26.26)	10.52 (8.92–12.12)	34.33 (32.20–36.45)
Widow/er	20.60 (15.92–25.28)	7.10 (5.13–9.07)	36.65 (33.22–40.08)
Divorced/Separated	21.88 (18.56–25.19)	8.13 (6.33–9.92)	38.33 (36.47–40.18)
Married	21.91 (21.28–22.53)	7.92 (7.56–8.29)	38.23 (37.84–38.62)
Single	23.24 (21.85–24.63)	9.24 (8.42–10.02)	36.06 (35.20–36.93)
p value	NS	NS	NS
Employment sector			
Public	22.24 (21.68–22.80)	8.26 (7.94–8.58)	37.59 (37.23–37.94)
Private	18.93 (15.46–22.40)	6.84 (4.78–8.90)	40.79 (39.16–42.42)
p value	NS	NS	<0.001
**Working in this department was my own choice**			
No	24.41 (23.57–25.24)	9.08 (8.59–9.57)	36.94 (36.43–37.45)
Yes	20.36 (19.65–21.07)	7.51 (7.10–7.92)	38.27 (37.80–38.74)
p value	<0.001^2^	<0.001^2^	<0.001^2^
**My job is stressful**			
No	16.96 (16.17–17.76)	7.09 (6.59–7.60)	38.82 (38.19–39.44)
Yes	24.77 (24.11–25.44)	8.78 (8.38–9.18)	37.08 (36.66–37.49)
p value	<0.001^2^	<0.001^2^	<0.001^2^
**Feeling of fatigue**			
Yes	22.82 (22.25–23.39)	8.32 (7.99–8.65)	37.66 (37.31–38.02)
No	14.04 (12.66–15.33)	7.10 (6.08–8.11)	38.33 (36.90–39.76)
p value	<0.001^2^	0.024	NS
**Your fatigue interferes the quality of your work?**			
Yes	25.95 (25.16–26.74)	9.69 (9.23–10.15)	36.36 (35.86–36.86)
No	18.16 (17.49–18.82)	6.63 (6.21–7.05)	39.16 (38.70–39.63)
p value	<0.001^2^	<0.001^2^	<0.001^2^
**Who helped you choose your profession?**			
Solely my decision	20.92 (20.24–21.60)	7.67 (7.28–8.06)	38.28 (37.86–38.69)
Circumstances (others decide on my behalf)	25.36 (24.02–26.71)	9.35 (8.57–10.14)	36.81 (35.93–37.69)
Encouraged by others	22.69 (21.40–23.99)	8.79 (8.02–9.57)	36.68 (35.84–37.51)
p value	<0.001	0.009	0.001
**I would dare to say that I suffer from emotional exhaustion**			
Totally disagree	16.42 ± 8.55		
Disagree	17.53 ± 8.26		
Moderately disagree	20.06 ± 9.19		
Moderately agree	24.05 ± 8.99		
Agree	30.58 ± 10.22		
Totally agree	33.97 ± 10.41		

^1^ANOVA test ^2^ *T*-test analysis.

### Self-reported fatigue among nurses and its relation with burnout

The prevalence of self-reported fatigue in nurses was 91.9%. The prevalence in the nurses who work in public sector was 92.4% as opposed to 82.2% in the private sector (p = 0.021). As seen in Table 4, two thirds of the nurses experienced fatigue very often or often. For half of the nurses fatigue worsens as time goes by and is mainly attributed to their job. Fatigue is mainly attributed to the work conditions; its nature is both physical and psychological and tends to appear after work.

**Table 4:** Characteristics of self-reported fatigue in the nurses

**Question**	**N**	**%**
**Do you feel fatigue?**		
No	118	8.1
Yes	1,340	91.9
**How often do you feel fatigue?**		
Very rarely	87	6.0
Rarely	345	23.6
Often	797	54.5
Very often	233	15.9
**I would say that the fatigue I feel:**		
Worsens as time goes by	688	47.5
Remains at the same levels every day	603	41.6
Improves as time goes by	157	10.8
**I would say that my fatigue is due to:**		
My work	794	55.0
My family	49	3.4
My health problems	49	3.4
All the above	423	29.3
Other	128	8.9
**My fatigue is:**		
Physical	167	11.6
Psychological	149	10.3
Mainly physical	170	11.8
Mainly psychological	177	12.3
Both physical and psychological	780	54.0
**When do you feel fatigue?**		
Mainly in the morning	108	7.7
Mainly at night	457	32.7
After work	831	59.5

Prevalence of fatigue was higher in females (93%) than in males (87.5%) (p = 0.003). Seventy three percent of the female nurses as opposed to 57% of the male nurses feel fatigue very often (p < 0.001). A higher percentage of male nurses (61.4%) than females (59.2%) experienced fatigue after work (p < 0.001). Statistically more male nurses (66.4%) that females (52.2%) attributed fatigue to their work conditions (p = 0.001). Our analysis showed that age did not influence fatigue (mean age of fatigued is 36.86 ± 10.40 versus 34.93 ± 9.99 in the non-fatigued). Those who feel fatigue mainly after their work were statistically older (37.47 ± 10.87) than those who replied mainly at night (36.03 ± 9.58) and mainly in the morning (33.13 ± 9.10). Mean work experience did not influence fatigue (p = 0.083). The nurses who stressed that their fatigue interferes with the quality of their services had less mean working experience (p < 0.001) and were younger (p < 0.001) than those who did not. As opposed to the burnout prevalence, fatigue prevalence did not differ among the departments where nurses work (p = 0.166) and among nurses with different marital status (p = 0.553).

The point prevalence of burnout was:

(1) 13.7% in those who feel fatigue as opposed to those who do not feel it (1.8%) (p < 0.001)

(2) 27.5% in those who feel fatigue very often, 13.4% in those who feel it often, 7.1% in those who feel it very rarely and 3.1% in those who feel it rarely (p < 0.001)

(3) 17% in those who believe that their job is stressful and 4.3% in those who do not believe it (p < 0.001)

(4) 18.6% in those who feel fatigue mainly in the morning, 15.8% who feel it mainly after work and 6.5% mainly at night (p < 0.001)

(5) 18% in those who replied that fatigue worsens as time goes by, 8.9% for those who replied that fatigue levels remain the same and 5.4% who replied that fatigue improves as time goes by (p < 0.001)

(6) 15.5% in those who believe that their fatigue is attributed to their work, 14.6% in those who believe fatigue is due to their family, 10.2% all the above factors, 9.1% to their health problems and 7.6% to other factors (p = 0.031)

(7) 27.7% in those who mentioned that their fatigue is psychological or mainly psychological, 14.9% both physical and psychological and 13% in those who replied that it is physical or mainly physical (p = 0.005)

(8) 75.4% in those who agreed with the statement I would dare to say that I suffer from emotional exhaustion and 20.2% in those who disagreed (p < 0.001)

(9) 20.8% in those who believed that their fatigue interferes the quality of their work and 3.9% in those who did not believe it (p < 0.001).

As seen in Table 3 those with fatigue rated more in the EE and the DP score. On the contrary the estimated odds of suffering from burnout was about 8.7 times higher among the nurses who did not report fatigue than among those who reported fatigue in this sample (OR, 8.70; 95%CI: 2.130–35.563). The relative risk of suffering from burnout was nearly 1.14 times as high for those who did not report fatigue as for those who reported it.

### Predictive validity of emotional exhaustion subscale

To test the predictive validity of the emotional exhaustion subscale nurses were asked to answer to the following question: “I would dare to say that I suffer from emotional exhaustion by choosing between totally disagree to totally agree” (6-item likert scale). As seen in Table 2, ANOVA test revealed that the emotional exhaustion subscale can predict very well the emotional burden of nurses, as those who totally agreed with the statement scored more on the EE subscale.

### Factors associated with burnout on regression analysis

In Table 5, Model 1 was devised to explore the effect of fatigue (Odds = 5.10, p < 0.003) on burnout after we have included “my job is stressful” and “age”, before we have introduced EE and DP to the model 1. It was found that feeling fatigue became insignificant factor (p > 0.05), as long as, we added EE and DP and their interaction to models 2 – 5. This was what we expected since the meaning of the measures of EE and DP have already accounted for the fatigue effect, which cannot co-exist with EE and DP in any of the models 2–5. It is worth noting that when we added the measures EE and DP, the deviance of the model reduced significantly. Most importantly, the interaction of EP and DP found to be highly significant in all models. Results confirmed that there is a variation within gender. Random effects were used to monitor the variation at the level of gender, gender within geographical location and fatigue effect within Gender. The ward choice, the sector (private or public) and the department had no significant effect to the burnout.

**Table 5:** Predictors of burnout by using binary logistic regression and of EE, DP and PA by using linear regression

**Independent variable**	**Model 1**		**Model 2**		**Model 3**		**Model 4**		**Model 5**	
	**Odds ratio(95%CI)**	**p-value**	**Odds ratio(95%CI)**	**p-value**	**Odds ratio(95%CI)**	**p-value**	**Odds ratio(95%CI)**	**p-value**	**Odds ratio(95%CI)**	**p-value**
**Fixed effect**										
My job is stressfulYes (1)	4.64(2.65 – 8.11)	<0.001	5.81(1.01–34.04)	0.05	4.14(1.28–13.40)	0.018	7.08(1.28–39.08)	0.02	6.65(1.21–36.47)	0.03
Age	0.96(0.94–0.98)	<0.001	0.94(0.89–0.94)	0.03	0.95(0.91–0.99)	0.014	0.94(0.89–0.99)	0.03	0.94(0.89–0.99)	0.04
Fatigue	5.10(1.22 – 21.43)	<0.003	7.63 (0.05–11.25)	0.42	4.96(4.37–5.63)	0.1964	-		-	-
EE	-		0.69(0.61–0.79)	<0.0001	1.38 (1.29–1.49)	<0.001	0.69(0.60–0.79)	<0.001	0.69(0.61–0.79)	<0.001
DP	-		0.18(0.11–0.31)	<0.0001	1.58 (1.43–1.76)	<0.0001	0.17(0.1–0.30)	<0.001	0.18(0.11–0.31)	<0.001
EE*DP	-		1.08(1.06–1.10)	<0.0001	-	-	1.08(1.05–1.11)	<0.001	1.08(1.06–1.10)	<0.001
**Random effect (variances)**										
Gender	0.0494		1.6474				1.48		0.0046458	
Gender (within town)Male (1)					0.918				0.067340	
Fatigue (within gender)							0.43		0.0050315	
Deviance	812.9		123.6		207		125.7		126.6	
Null model deviance = 953										

## Discussion

This research has explored the factors associated with nurses’ self-reported fatigue correlated with the burnout syndrome. The strength of the study was its representativeness, since 53% of the total nurses’ population in Cyprus was surveyed across all clinical settings and geographical regions. To the best of our knowledge this is the first published nationwide research of its kind in Cyprus. The implications in practice include but not limited to the development of a national action plan for the prevention and the management of burnout syndrome in Cypriot nurses in the clinical settings. The study also aimed at exploring the relation between self-reported fatigue and burnout syndrome which is also of particular interest to the international literature.

The health care clinical settings are a highly stressful environment and may therefore be associated with a high rate of burnout syndrome and fatigue especially when it comes to nurses [49,50]. Yoder for example in a combined quantitative and qualitative study in nurses working in various clinical settings concluded that highly stressful environments are considered as triggers for burnout and fatigue [22]. Similarly, Maytum et al. [51] in a descriptive qualitative study of 20 nurses working in pediatric ward claimed that the nature of the environment and the type of patients needing care were a source of fatigue and burnout. However, preceding studies have revealed an apparent paradox that of a low degree of burnout in high stress health care environments [34,41,45,49,50]. This research coincides with these studies, contributing to the paradox that even though nurses acknowledge their work as stressful at the same time they report average to low degree of burnout. An average degree of burnout is reflected in average scores on the three subscales, and a low degree of burnout is reflected in low scores on the EE and DP subscales and a high score on the PA subscales [8,43]. A low degree of burnout therefore represents a positive psychological condition rather than the stereotypical negative condition that is widely associated with the burnout syndrome.

A total of 12.8% of the participating nurses met the Maslach’s criteria for a high degree of burnout. According to Maslach et al. [8] a high degree of burnout is reflected in high scores on the EE and DP subscales and in low scores on the PA subscale which is rated inversely. This finding indicates the correlation between stressful working environments with high degree of burnout. This is consistent with the body of literature that supports this relationship [51-55].

The analysis demonstrated that the percentage of nurses with high EE was 21.5%, a finding which mainly reflects the organizational and the social climate of the work environment according to Maslach et al. [43] and Yoder [22]. A possible interpretation of this finding might reflect the nurses’ higher ability to adapt to the demands of their clinical setting as opposed to the findings of other studies [56,57].

What has been stressed by earlier studies [53-55,58-60] that the type of ward plays an important role as to the expressed levels of burnout has also been demonstrated by this study. The levels of burnout reported by the participants varied accordingly. Nurses in the oncology departments, for example expressed the highest levels of burnout (21.9%) compared to their colleagues working in operating theatres (17.5%), in surgical wards (17.2%) and in the emergency departments (15.9%). This burnout pattern was also supported in the Yoder study [22] demonstrating that the nature of the clinical environment (i.e. ward type) as well as the type of cases that require care can pose an influence on the levels of burnout experienced by the nurses.

In contrast to the earlier findings, the researchers found relatively low expressed levels of burnout among nurses working in the ICU units. Whilst prior work [57,61,62] expected that ICU environments would be highly stressful and potentially burnout generating, this study showed that the nurses in Cyprus working in such environments do not necessarily express higher levels of burnout compared to colleagues working in other clinical settings. This finding can partially be explained by a number of possible reasons routed in the context of the clinical settings in Cyprus. Such reasons for example can be the type of cases cared; the amount of training received the staffing levels, the working conditions and the psychological support services available to the Cypriot nurses.

The researchers anticipated that the employment type (private vs. public sector) would have an effect on the reported burnout levels reported in this study. Their expectations were based on the fact that the nurses working in the public sector tend to enjoy better working conditions (i.e. better salary, less working hours, permanent status of employment) compared to those who are employed in the private sector. Paradoxically nurses who work in the private sector reported lower feelings of EE and overall burnout than their colleagues in the public healthcare settings (12.7% of those who work in the public sector and 12.2%). This finding can possibly be interpreted by the fact that recent changes in the national health care system in Cyprus have positively influenced some (if not all) of the perceived disadvantages in the private sector. These changes have been implemented not only as a means to increase the quality of the provided care but also to bring equilibrium between private and public healthcare sectors. The improvement in the working conditions in the private sector was also reflected on the levels of fatigue experienced by the nurses. Another issue that potentially contributed to this finding is the fact that nurses in the private sector only provide secondary care and some preventative services. Statistical analysis showed that the fatigue prevalence in the nurses who work in public sector was 92.4% as opposed to 82.2% in the private sector. The employment sector also affected the level of PA with those who worked in the public sector having lower mean PA score. This can possibly be explained by the fact that in the public sector there are less feedback mechanisms and personal accomplishments strategies in place compared to the private sector.

An important aim of this paper was to clarify whether the burnout syndrome and fatigue experienced by the nursing staff might somehow be related. This is an area that received scarce attention in the literature and therefore the findings of this study are new to the relative literature. The researchers prior the study expected that a correlation between these two variables would exist and perhaps be explained by the stressful environments in which nurses’ work [63]. A few studies [64-66], support the association between fatigue and stress. Indeed, a common finding that might offer an acceptable interpretation to the above expectation is the fact that nurses acknowledging that their job is stressful appear more susceptible to burnout and self-reported fatigue. The point prevalence for fatigue was 17% in those who believed that their job was stressful and 4.3% in those who do not believe it indicating that it is more likely that this group of nurses will experience fatigue compared to their colleagues that do not see their job as stressful. This point of prevalence is consisted with those of earlier studies [67]. Through the multilevel logistic regression analysis “my job is stressful” is a significant predictor of burnout onset. Asking the nurses to respond to this question could be an indirect predictor of their burnout.

The analysis demonstrated that burnout is correlated with EE and DP, with females being more susceptible [68,69]. Perhaps the factor that explains this phenomenon is that women often have a double and possibly conflicting role, namely the one of the healthcare professional and the other of the mother (and/or housekeeper); this may increase their levels of stress and drains their energy overall [70].

This study has provided new insights into the nature of the relationship between the type of organization (private or public) and the type of ward (medical, surgical, oncology), nurse burnout, nurse self-reported fatigue, and the link between nurse burnout and nurse self-reported fatigue, however further research in the future will be needed to more fully understand the causal mechanisms that link these and other organizational features and outcomes.

The study presents several limitations especially regarding its generalisability. First, Cyprus may differ regarding factors associated with burnout syndrome and fatigue, such as relationships between physicians and nurses. However, our sample was large and representative of different types of nursing wards. The large difference in the sample size of nurses working in the public and the private sector should be taken into account. The study seems to be more representative for the public rather than for the private sector. The analysis did not take into consideration possible confounders. Another limitation of the study was that the analysis did not take into consideration the various levels (ranks) of nurses included in the study. One limitation that was attributed to the demographic details acquired by the nurses was the fact that only specific clinical settings were provided as option whilst the other settings were merged into one category namely the “other”. As a result no further information could ne attained on the nature of these other settings.

This study in the future could be methodologically improved by attempting to measure the incidence rate of burnout among nurses in the various participating department. Perhaps a way of doing this would be through a series of basic self-report questions regarding the onset of fatigue and burnout in a correlational design. In conclusion any future studies on the topic under investigation should consider issues such as the hours of work per shift, hours of work per week, voluntary or mandatory overtime, the days off per week as well as other workload measures. Based on the human factors models of Carayon and Gurses [49] and Karsh et al. [71] these measures fall into three types of workload namely the unit-level workload, the job-level workload and the task-level workload. These variables might have a different impact on outcomes such as quality of care, patient safety, nurse job dissatisfaction and burnout.

## Conclusions

The results of this study indicate that burnout and fatigue are constructs that can be attributed to the stressful clinical settings in which nurses in Cyprus work in. With two thirds of the nurses experiencing fatigue very often or often and with twenty one point five percent of the participants were in the high EE range, 30.7% scored high in the PA section and 33% scored high in the DP section of the scale, it is clear that the problem affects a high percentage of the nursing population in Cyprus. High burnout scores are more likely to be associated with certain variables such as: fatigue, age and job-related stress. Further research however is needed to explore the problem in depth as well as to develop intervention and supporting programs for the nurses that are effective. Analyzing the prevalence of burnout and fatigue within a healthcare organization is an essential first step for organizations that aim to implement stress reduction programs and establish positive work environments for their workers.

## Competing interests

The authors declare that they have no competing interests.

## Authors’ contributions

VR conceived, designed, acquired the data, coordinated the study, wrote, edited, revised the manuscript, analyzed the data and interpreted the results; AC contributed to acquiring the data, writing of the background, the discussion, the conclusions and revising the manuscript. MT contributed to the statistical revision of the manuscript. All authors read and approved the final manuscript.

## Pre-publication history

The pre-publication history for this paper can be accessed here:

http://www.biomedcentral.com/1471-2458/12/457/prepub
